# Case Report: Asymmetric recovery unmasking watershed infarction in a patient with ICU-acquired weakness

**DOI:** 10.3389/fmed.2026.1858632

**Published:** 2026-06-24

**Authors:** Bingqing Zhao, Meng Yu, Peining Zhou, Fan Li, Junfang Huang, Jing Ma, Zhaoxia Wang

**Affiliations:** 1 Department of Respiratory and Critical Care Medicine, Peking University First Hospital, Beijing, China; 2 Department of Neurology, Peking University First Hospital, Beijing, China

**Keywords:** case report, critical illness polyneuromyopathy, ICU-acquired weakness, ischemic stroke, watershed infarction

## Abstract

**Background:**

Intensive Care Unit-acquired weakness (ICUAW) typically manifests as generalized, symmetrical flaccid paralysis. However, atypical asymmetric patterns can occur when concurrent neurological complications are present, posing a significant diagnostic “double-masking” challenge in critically ill patients.

**Case presentation:**

A 59-year-old female with severe pneumonia, septic shock, and complex endocrine comorbidities developed profound muscle weakness following survival from acute respiratory failure. Initially, the generalized weakness was attributed to ICUAW, supported by electrophysiological studies and a muscle biopsy. However, the patient exhibited distinct asymmetrical motor recovery, with persistent left-sided deficits despite a negative cranial computed tomography (CT). Subsequent cranial magnetic resonance imaging (MRI) revealed fresh watershed infarctions in the right hemisphere. A tailored rehabilitation strategy focusing on mitochondrial metabolism and active physical therapy led to significant functional improvement, with full recovery reported at a 2-year follow-up.

**Conclusion:**

This case highlights that the weaning process can act as a hemodynamic “stress test,” unmasking latent cerebrovascular fragility in complex ICU patients. Clinicians should maintain a high index of suspicion for asymmetric recovery patterns in ICUAW. A multimodal approach is essential for accurate diagnosis and management.

## Introduction

Intensive Care Unit-acquired weakness (ICUAW) is a complication of critical diseases, including critical illness myopathy (CIM), critical illness polyneuropathy (CIP), and critical illness polyneuromyopathy (CIPNM), mainly manifested by muscle weakness and difficulty in weaning from mechanical ventilation (1). The disease has a high occurrence in Intensive Care Unit (ICU) and the inconspicuous clinical manifestations of ICUAW, especially in patients who are sedated or unconscious, lead to it being frequently underdiagnosed due to sedation-masked neurological signs. The three subtypes of ICUAW are also difficult to be distinguished just depending on clinical manifestations (2), and their differentiation mainly depends on electrophysiological examination and muscle biopsy (1). ICUAW has no specific treatment, and its prognosis is not significantly related to its classification, consequently, muscle biopsy is seldom utilized in routine clinical practice. But sometimes, certain underlying neuromuscular diseases often need to be identified from obvious ICUAW by histopathology.

Here, we reported a critically ill patient with severe pneumonia and other relative comorbidities who developed severe weakness after survival from acute respiratory failure and mechanical ventilation. Muscle biopsy and pathological examination were made in this patient to investigate the causes of her profound muscle weakness and further confirmed the diagnosis of ICUAW.

## Case presentation

A 59-year-old female presented to our hospital with severe pneumonia and septic shock. Her medical history was intricate: 2 months prior to admission, she developed progressive lower extremity weakness and edema following a respiratory infection. One month before admission, she was diagnosed with anterior pituitary insufficiency and diabetes mellitus at another facility. Despite replacement therapy with levothyroxine and various regimens of corticosteroids–including intravenous hydrocortisone–her weakness progressed until she was wheelchair-bound. Two weeks before her current admission, a fever prompted a lumbar puncture at an external hospital to rule out central nervous system infection or Guillain-Barré syndrome; the results showed a pressure of 220 mmH2O but normal protein and cytology, effectively ruling out albuminocytological dissociation. Upon transfer to our ICU, her condition deteriorated rapidly, necessitating endotracheal intubation and mechanical ventilation.

To manage severe patient-ventilator asynchrony and facilitate lung-protective ventilation, a multimodal sedation and neuromuscular blockade (NMBA) regimen was implemented. This included midazolam (up to 10 mg/h) and fentanyl, alongside a 32-h continuous infusion of rocuronium bromide (5–6 μg/kg/min). While vasoactive support was successfully discontinued on the second day, the patient regained consciousness on the third day post-sedation. Although awake and interactive, she exhibited profound, generalized muscle weakness, unable to lift her limbs against gravity. The weaning process was prolonged for 2 weeks due to persistently high ventilatory parameters. On the first day post-extubation, neurological examination revealed a symmetric pattern of weakness: proximal muscle strength was Grade I–II, while distal strength was Grade III–IV across all four limbs. Crucially, her cranial nerve functions were fully preserved, evidenced by full extraocular movements and the absence of facial palsy. Furthermore, muscle tone was globally decreased, deep tendon reflexes were symmetrically diminished or absent, and bilateral pathological reflexes (e.g., Babinski sign) were negative.

The diagnostic process initially focused on three primary domains to investigate the etiology of her profound weakness:

- Drug-Induced and Metabolic Factors: On admission, normal creatine kinase levels ruled out rhabdomyolysis. While lactate dehydrogenase peaked at 1317 IU/L, its decline during anti-infective therapy alongside the absence of systemic symptoms excluded lipid storage diseases. Although laboratory tests confirmed HPA axis suppression (adrenocorticotropic hormone 4.17 pg/mL, cortisol 49.89 μg/dL), steroid-induced myopathy was unlikely to be the primary cause as the weakness predated steroid administration. Regarding her endocrine status, replacement therapy had successfully normalized thyroid-stimulating hormone and free thyroxine levels; however, free triiodothyronine remained mildly decreased (2.69 pmol/L). Crucially, her motor deficits showed no improvement following these hormonal adjustments, suggesting that hypothyroidism was not the primary driver of her current symptoms. Furthermore, aggressive electrolyte replacement to maintain normokalemia failed to improve motor function. Diabetic muscle infarction was excluded due to the absence of acute pain or swelling.
- Systemic and Pre-existing Comorbidities: Initial tumor markers were non-specifically elevated, but a subsequent positron emission tomography–computed tomography was negative, ruling out paraneoplastic syndromes. Negative results for M-protein and autoimmune spectra further ruled out primary autoimmune neuromuscular diseases.
- Acute ICU-Acquired Neuromuscular Complications: The combination of septic shock, prolonged mechanical ventilation, and exposure to NMBA and corticosteroids created a high-risk environment for ICUAW. Electrophysio- logical studies (Table 1) demonstrated generalized reduction in compound muscle action potential (CMAP) amplitudes with preserved conduction velocities, consistent with critical illness myopathy/polyneuropathy (CIM/CIP). Crucially, a left biceps brachii biopsy provided definitive evidence, revealing grouped small angular atrophic fibers and specific, diffuse atrophy of Type II fast-twitch fibers (Figure 1).

**TABLE 1:** Summary of electromyography (EMG) and nerve conduction study (NCS) findings.

A. Summary of nerve conduction studies.						
Nerve type	Nerve	Side	Latency (ms)	Amplitude (mV/μV)	Velocity (m/s)	Interpretation
Motor	Median	L	4.91	0.10↓	49.7	Axonal damage/myopathy
		R	4.92	0.12↓	50.5	
	Ulnar	L	9	0.56↓	49.5	Axonal damage/myopathy
		R	7.63	1.02↓	73.2	(Less severe than left)
	Tibial	L	16.6	0.53↓	36.2	Axonal damage/myopathy
	Peroneal	L	5.93	0.50↓	49	Axonal damage/myopathy
Sensory	Median	L	2.63	49.3	41.3	Mild slowing
		R	1.54	2.4↓	45.2	Axonal damage
	Ulnar	L	3.22	4.2↓	37.3	Axonal damage
		R	2.9	13.5	39.8	
	Sural	L	2.33	12.0	36.7	Normal/mild slowing
		R	2.83	7.6	44.2	Normal
**B. Summary of electromyography (EMG) findings.**						
**Muscle**		**Side**	**Spontaneous activity (Fib/PSW)**	**MUAP morphology and recruitment**		**Interpretation**
Sternocleidomastoid		Left	None	Normal duration, polyphasic		Myopathic changes
Biceps brachii		Right				
Abductor pollicis brevis		Right				
Tibialis anterior		Right	Present (+++)	Reduced recruitment (loss of MU)		Neurogenic/mixed pattern
Vastus medialis		Left	Present (++++)	No active recruitment (electrical silence)		Severe denervation/myopathy
Paraspinal (T10)		Right	None	Normal		Normal

↓indicates values below the lower limit of normal. Motor amplitudes are in mV; sensory amplitudes are in μV. The data demonstrates generalized reduction in compound muscle action potential (CMAP) amplitudes with relatively preserved conduction velocities, consistent with critical illness myopathy/polyneuropathy. Fib, Fibrillation potentials; PSW, Positive Sharp Waves; MUAP, Motor Unit Action Potential; MU, Motor Unit. Findings: EMG reveals abundant spontaneous activity in lower limb muscles and myopathic changes (polyphasia with normal duration) in upper limb and neck muscles, supporting a diagnosis of critical illness myopathy superimposed with neuropathy.

**FIGURE 1:**
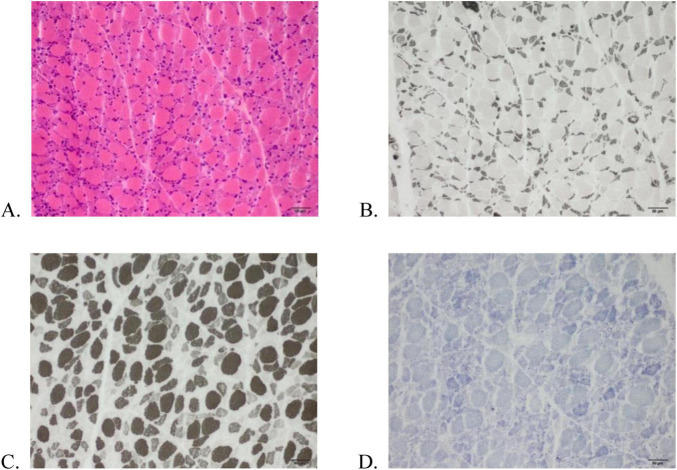
Muscle biopsy findings confirming CIM. **(A)** H&E stain (×200) showing grouped small angular atrophic fibers. **(B,C)** ATPase stain showing specific, diffuse atrophy of Type II fast-twitch fibers (dark in pH 9.4, light in pH 4.3). **(D)** SDH stain (×200) demonstrates marked unevenness in staining intensity with reduced enzyme activity in some Type II fibers.

The diagnostic narrative shifted on the 12th day post-extubation. While the right-sided power showed a steady trajectory of recovery, the left side remained recalcitrant, revealing a stark asymmetry that challenged the classic symmetrical paradigm of ICUAW. Physical examination revealed a distinct mixed pattern of upper and lower motor neuron signs:

- Cranial Nerves: While extraocular movements were full and no facial or lingual palsy was evident, horizontal nystagmus was observed on right lateral gaze.
- Motor System: There was generalized hypotonia and areflexia (absent deep tendon reflexes) in all four limbs. Muscle strength assessment revealed severe asymmetric weakness, significantly worse on the left side and in proximal muscles (Table 2A).
- Sensory: Subjective light touch was diminished in the left upper limb, and vibration sensation was reduced distally in the lower limbs.

**TABLE 2:** Muscle strength of each muscle group in the patient.

A. Muscle strength of each muscle group in the patient on the 12th day post-extubation.		
Muscle group	Left MRC score	Right MRC score
Shoulder abductor (deltoid)	1	2
Elbow flexor (biceps brachii)	1	3
Wrist extensor (extensor carpi radialis longus/brevis)	2	3
Hip flexor (iliopsoas)	1	3
Knee extensor (quadriceps femoris)	1	3
Ankle dorsiflexor (tibialis anterior)	1	2
**B.** **Muscle strength of each muscle group in the patient at discharge.**		
**Muscle group**	**Left MRC score**	**Right MRC score**
Shoulder abductor (deltoid)	1	3
Elbow flexor (biceps brachii)	1	4
Wrist extensor (extensor carpi radialis longus/brevis)	3	4
Hip flexor (iliopsoas)	1	3
Knee extensor (quadriceps femoris)	1	3
Ankle dorsiflexor (tibialis anterior)	2	4

A retrospective review of the initial electrophysiological data revealed a subtle asymmetry: the left ulnar nerve motor amplitude was disproportionately lower (0.56 mV) than the right (1.02 mV). Initially, this localized finding was overshadowed by the patient’s severe generalized flaccidity and a muscle biopsy confirming CIM. This stark asymmetry–deviating from the typical symmetric recovery of CIM–prompted a cranial MRI (Figure 2). The scan revealed multiple fresh watershed infarctions in the right corona radiata, basal ganglia, and medial frontal lobe. These lesions involved corticospinal projection fibers, explaining the contralateral motor deficit. Subsequent vascular assessment identified subtotal occlusion of the right internal carotid artery (ICA) and severe right vertebral artery stenosis. The patient had no history of hypertension, smoking, or premature atherosclerotic disease. The absence of routine medical check-ups likely allowed her underlying endocrine and vascular comorbidities to progress asymptomatically until clinical decompensation occurred.

**FIGURE 2:**
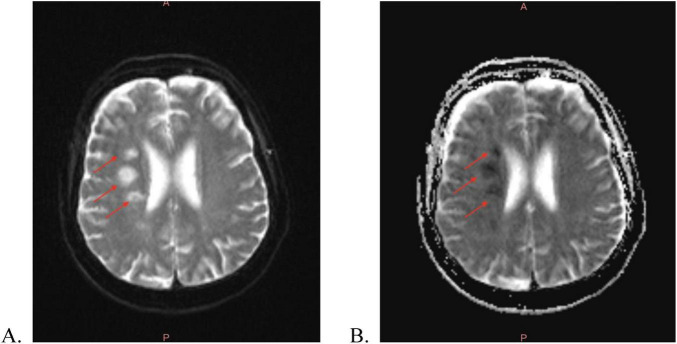
Neuroimaging revealing the cause of asymmetry. **(A)** Diffusion-Weighted Imaging (DWI) and **(B)** ADC map showing restricted diffusion consistent with fresh watershed infarctions (arrows) in the right corona radiata and basal ganglia, explaining the patient’s left-sided weakness.

Given the new-onset cerebral infarction, antiplatelet and plaque-stabilization therapies were initiated. We implemented a tailored strategy focusing on mitochondrial energy metabolism (idebenone, coenzyme Q10) and early active physical rehabilitation. At discharge, which was 1 month after the initial onset of weakness in the ICU, the patient’s total Medical Research Council (MRC) score had improved to 30, with muscle strength partially recovered as shown in Table 2B.

During a subsequent follow-up via telephone, the patient reported a complete subjective recovery of muscle strength, which was achieved approximately 2 years after discharge. The patient has regained full functional independence in daily activities and is now capable of engaging in strenuous physical exercise, including brisk walking and mountain climbing, with no perceived discrepancy in bilateral limb power. The overall timeline of the patient’s clinical course, from pre-admission presentation to the 2-year follow-up, is summarized in Figure 3.

**FIGURE 3:**
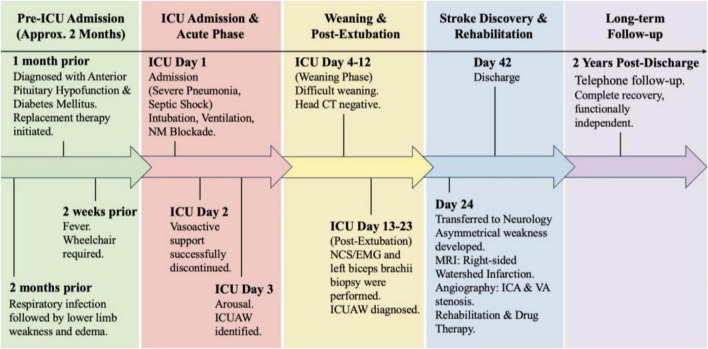
Timeline of clinical events. This figure illustrates the chronological sequence of the patient’s pre-admission history, acute ICU management, diagnostic workup for ICUAW, and the subsequent discovery of the concurrent watershed cerebral infarction, concluding with the long-term follow-up.

## Discussion

The patient’s 2-months history of weakness, coupled with HPA axis suppression and hypothyroidism, created an initial “diagnostic fog.” Differentiating between the progression of chronic endocrine myopathy and acute sepsis-induced injury was the first hurdle. The diagnosis of ICUAW was only secured after aggressive hormonal replacement failed to improve motor function and was definitively confirmed by the muscle biopsy showing specific Type II fiber atrophy. This underscores the necessity of histopathological evidence in complex patients where pre-existing morbidities mimic ICUAW.

Intensive Care Unit-acquired weakness is canonically characterized by diffuse, symmetric flaccid paralysis that typically spares the cranial nerves (3). In the present case, the diagnostic landscape was significantly confounded by the patient’s intricate medical history and the presence of lower limb weakness preceding ICU admission. Furthermore, the pervasive and generalized motor deficits characteristic of ICUAW created a “background noise” that effectively overshadowed the focal manifestations of the superimposed cerebral watershed infarction. Consequently, the underlying central nervous system insult only became clinically discernible through the emergence of asymmetric motor recovery during the rehabilitation phase. However, retrospectively, the distinct left-right asymmetry in the early electrophysiological data may have provided a crucial preliminary clue.

The pathogenesis of ICUAW is multifactorial, with established risk factors including severe sepsis, multi-organ failure (4), and prolonged mechanical ventilation (5). Sepsis acts as the primary insult, triggering systemic inflammation and mitochondrial dysfunction that leads to ICUAW through selective type II fiber atrophy (6).

While hemodynamic stability was restored in the short term, the negative cranial CT in the second week–performed just prior to extubation–and the subsequent evidence of fresh infarction on the third-week MRI supports the hypothesis that the stroke likely occurred during the transition/weaning phase, though this temporal inference remains speculative in the absence of direct real-time hemodynamic evidence (7). The physiological stress of spontaneous breathing trials (SBTs) acutely increases cerebral metabolic demand and triggers sympathetic surges. In the context of pre-existing carotid stenosis and sepsis-impaired autoregulation (8), this “stress test” exhausted the patient’s limited hemodynamic reserve. Even in the absence of overt systemic hypotension, the preceding septic insult had critically compromised cerebral perfusion pressure; consequently, localized flow in vulnerable border zones plummeted below the ischemic threshold (9). This underscores that for complex ICU patients, the weaning process can unmask latent cerebrovascular fragility long after systemic stability is perceived to have been regained. However, we must acknowledge an inherent limitation of our report: in the absence of direct cerebral hemodynamic measurements during the weaning process, this causal link rests primarily on temporal inference derived from the sequential neuroimaging findings.

The incidence of septic shock is reported to be over 6% (10), creating a high-risk environment for perfusion-related complications. Particularly, patients with underlying cardiovascular and cerebrovascular diseases or multiple risk factors are more prone to develop watershed infarction due to hypoperfusion during ICU stay (11). Critically ill patients lose nearly 2% of their skeletal muscle mass daily during the first week of ICU admission (12), and approximately 40% of critically ill patients develop ICUAW (13). However, reports documenting ICUAW superimposed with cerebral watershed infarction are virtually nonexistent. This paucity of data highlights the diagnostic challenge: detecting a concurrent ischemic stroke within the context of diffuse critical illness weakness demands a high index of clinical suspicion to navigate the diagnostic overshadowing of ICUAW.

Definitive resolution of this complex clinical scenario necessitated a multimodal approach. Cranial MRI was indispensable to elucidate the structural basis of the asymmetry, uncovering the occult watershed infarctions missed by CT (7). Equally critical was the role of muscle biopsy, particularly given the patient’s complex medical background. Non-invasive electrophysiological studies failed to distinguish between these possibilities. Uniquely, the biopsy provided the decisive evidence: the finding of selective type II fiber atrophy is the pathognomonic signature of CIM (1), distinct from chronic metabolic myopathies. Furthermore, although steroid-induced myopathy also predominantly affects Type II fibers, it usually manifests as scattered atrophy without the organized, grouped angular patterns or the profound bioenergetic failure (as evidenced by our SDH staining) typically seen in CIM (1, 14). Crucially, the biopsy allowed us to exclude metabolic and endocrine myopathies–such as those secondary to the patient’s pre-existing anterior pituitary insufficiency. Metabolic myopathies often present with non-specific changes or lipid/glycogen accumulation, which were absent in our histopathological evaluation (4).

Although the initial muscle biopsy did not demonstrate extensive neurogenic pathological changes, the integration of clinical examination, nerve conduction studies, and electromyography provided robust evidence for a concurrent neuropathic process. Consequently, the final diagnosis was established as CIPNM, characterized by both myopathic and neuropathic components.

We postulate that the patient’s pre-admission lower extremity weakness was a non-specific constitutional symptom associated with the acute respiratory infection, rather than a focal neurological deficit. Thus, histopathology was essential not merely for diagnosis, but to disentangle acute ICU-acquired pathologies from the patient’s complex baseline of chronic comorbidities.

Regarding functional recovery, early implementation of physical rehabilitation is universally advocated (15). While adjunctive neuromuscular electrical stimulation (NMES) has been shown to enhance local muscle strength, shorten the duration of mechanical ventilation, and potentially prevent ICUAW (16, 17), its broader clinical efficacy remains debated. Current evidence does not robustly support its ability to improve overall functional status at discharge, accelerate awakening, or reduce mortality (16). Furthermore, its clinical utility is frequently limited by sensory discomfort and poor patient tolerance (18). Given these limitations and the presence of a superimposed stroke, we eschewed NMES for this patient. Instead, we implemented a tailored strategy comprising early, active physical rehabilitation combined with targeted pharmacotherapy to improve mitochondrial energy metabolism. This approach directly addresses the bioenergetic failure inherent to critical illness myopathy (14) while simultaneously providing well-established benefits for motor neurorehabilitation following ischemic stroke (19), thereby comprehensively addressing both facets of her dual pathology.

Crucially, this case highlights the shared hemodynamic vulnerability of both the central and peripheral nervous systems. Strict hemodynamic optimization–specifically the rapid correction of hypotension–is therefore paramount not only to mitigate the microcirculatory failure driving ICUAW, but also to prevent border-zone cerebral ischemia in patients with underlying vascular risk factors.

Regarding the long-term prognosis, the synergistic nature of this “dual-hit” injury suggests a more protracted and challenging recovery course compared to isolated ICUAW. While patients with ICUAW alone often show gradual improvement over months (20), the superimposed cerebral watershed infarction in this case introduces permanent structural damage to the upper motor neurons (19). As evidenced by the limited improvement in the patient’s left-sided MRC scores at discharge, long-term functional recovery will likely be constrained by the severity of the stroke. Future management should focus on a multi-stage rehabilitation trajectory, transitioning from intensive inpatient therapy to community-based support (21), with realistic goals focused on maximizing compensatory strategies and preventing secondary complications.

## Limitations

Our study has several limitations. First, the patient’s long-term follow-up was conducted via telephone, which precluded objective neurological examination or follow-up neuroimaging. Second, the temporal link between the weaning process and the occurrence of watershed infarction remains a hypothesis based on sequential imaging findings; due to the absence of continuous, direct cerebral hemodynamic monitoring, we cannot definitively establish causality. Finally, while the histopathological findings definitively confirmed the myopathic component of the patient’s weakness, the inherent limitations of muscle biopsy sampling may have resulted in an underestimation of the concomitant neuropathic pathology.

## Conclusion

This case underscores that in critically ill patients with complex medical backgrounds, the etiologies of muscle weakness are diverse. Therefore, a high degree of vigilance should be maintained for atypical or asymmetric limb weakness. A multimodal approach, using biopsy to define the generalized pathology and neuroimaging to rule out focal complications, is essential to disentangle superimposed conditions and ensure precise management.

## Data Availability

The original contributions presented in this study are included in this article/supplementary material, further inquiries can be directed to the corresponding author.
